# Experiences in Improving the Quality of Community-Based Fever Management from Three Malaria-Endemic African Countries

**DOI:** 10.4269/ajtmh.23-0488

**Published:** 2024-01-09

**Authors:** Jehan Ahmed, Adam Nothem, Jadmin Mostel, Annie Ciceron, Luigi Nuñez, Oméga Raobela, Andry Patrick Raoiliarison, Sosten Lankhulani, John Munthali, Mady Cissoko, Beh Kamaté, Oumar Yattara, Samba Coumaré, Katherine Wolf

**Affiliations:** ^1^ PMI Impact Malaria, Population Services International, Washington, District of Columbia;; ^2^ Programme National de Lutte contre le Paludisme, Antananarivo, Madagascar;; ^3^ PMI Impact Malaria, Population Services International, Antananarivo, Madagascar;; ^4^ National Malaria Control Program, Lilongwe, Malawi;; ^5^ PMI Impact Malaria, Population Services International, Lilongwe, Malawi;; ^6^ Programme National de Lutte contre le Paludisme, Bamako, Mali;; ^7^ PMI Impact Malaria, Population Services International, Bamako, Mali;; ^8^ PMI Impact Malaria, Jhpiego, Washington, District of Columbia

## Abstract

The WHO affirms that trained, supervised, and supported community health workers (CHWs) can deliver high-quality health services effectively and has called for documentation of enabling factors, needs, and implementation strategies of successful CHW programs. In response, the U.S. President’s Malaria Initiative Impact Malaria Project conducted a study to document implementation approaches, best practices, and lessons learned for quality improvement (QI) of community-based fever management in Madagascar, Malawi, and Mali. The team conducted 10 key informant interviews (KIIs) with individuals at national, regional, and district levels using an open-ended interview guide tailored to each level, and a desk review of documents and materials related to community-based QI. Each country’s community health landscape and QI approaches were summarized into four categories identified during the KIIs (training, supervision, coaching/mentoring, and review meetings) and compared. Results found that Madagascar, Malawi, and Mali all had well-defined community health strategies that include QI, but countries could not extend their full package of community-based QI approaches to all CHWs as a result of limited human and financial resources. Vertical funding for health programs limits the scope and coverage of QI approaches, especially at the community level. Recommendations from key informants for strengthening community-based QI included integrating QI approaches to improve cost efficiency, to define roles and responsibilities more clearly, to engage communities and all health system levels in implementation, and to digitize QI tools. Increased financial and skilled human resources are needed for community-based QI activities to achieve their intended effect.

## INTRODUCTION

More than 5 million children died before reaching their fifth birthday in 2021.^1^ Pneumonia, diarrhea, and malaria—all preventable and treatable diseases—remain the main causes of child mortality, accounting collectively for 32% of annual deaths among children younger than 5 years in 2019.^2^ Globally, 54 countries are not on track to achieve the Sustainable Development Goals (SDGs) for the child survival target of fewer than 25 deaths per 1,000 live births by 2030.^1^

Reaching the child health–related SDG targets requires strong primary health-care systems, including the institutionalization of high-quality health care delivered at the community level through the work of community health workers (CHWs).^3^ The WHO has consolidated evidence indicating that properly trained, supervised, and supported CHWs can deliver a range of preventive, promotive, and curative health services effectively.^4^ Investments in CHWs and the services they provide help fill gaps in facility-based care, improve access to health care, and save lives.

The WHO and UNICEF recommend the integrated community case management (iCCM) strategy to train and supervise CHWs to provide life-saving care for malaria, pneumonia, and diarrhea by targeting hard-to-reach, vulnerable populations through the rational use of medications and the promotion of nutrition, timely care seeking, and referral of severe cases to higher levels of care.^5^^–^^8^

The WHO defines quality improvement (QI) of health service delivery as “an approach to the improvement of service systems and processes through the routine use of health and program data to meet patient and program needs (p. 282).”^9^ Other definitions in the literature align well with that of the WHO, emphasizing a systematic approach to improving service provision and patient outcomes by strengthening institutional knowledge, skills, and infrastructure.^10^^–^^15^ However, the WHO places additional emphasis on using data to drive QI, which includes measuring impact and changes over time, and understanding the variation in processes and outcomes.^11^ Community health workers can be supported by digital tools, which have been shown to improve the access, quality, and collection of CHW performance data to inform supervision and allow real-time problem solving and action planning. These digital tools can also provide a platform for CHW training, peer learning and engagement, skills building, and on-demand learning.^16^^–^^19^ In addition, the integration of data from community-based care in surveillance systems creates new opportunities and challenges for tracking the impact of disease control and child survival initiatives.^20^^,^^21^

The WHO has identified a need for comprehensive case studies that document the critical components of successful CHW programs, including enabling contextual factors, health system needs, and the challenges and opportunities of implementing several interventions simultaneously.^4^ Community health worker programs and policies need to be monitored over time and adapted using context-specific evidence. As such, policymakers and managers are encouraged to share data on the characteristics of CHWs and their performance, and information on program implementation and effectiveness.^22^^,^^23^

As the flagship global malaria service delivery project of the U.S. President’s Malaria Initiative (PMI), the PMI Impact Malaria Project supports national malaria programs (NMPs) to improve the quality of health service delivery through approaches such as facility-level Outreach Training and Supportive Supervision (OTSS); mentorship; peer-to-peer learning; and targeted classroom training for providers.^24^ To address the CHW program documentation gap identified by the WHO and to reflect on feasible and effective approaches for measuring and improving the quality of service delivery, PMI Impact Malaria conducted a study to describe the QI approaches used in three malaria-endemic African countries to improve community-based fever management and share experiences from their implementation.

## MATERIALS AND METHODS

This study was conducted to document experiences from each country’s implementation of community-based QI. It was not designed to assess program effectiveness or compare country QI approaches and their implementation. The study team conducted country case reviews from September to December 2022 in Madagascar, Malawi, and Mali. Of 19 countries receiving support from the PMI Impact Malaria Project, these three countries met the following study selection criteria: a functioning CHW program in a significant part of the country between 2018 and 2021, linguistic and geographic representation, and ability to secure ethical approval within a defined time frame. The study team completed the analysis in two parts: semistructured qualitative key informant interviews, and a desk review of existing documents and materials.

### Key informant interviews.

Semistructured, qualitative key informant interviews were conducted with national- and subnational-level health authorities in each country to understand more fully the implementation and monitoring of QI activities, program successes and challenges, and lessons learned. Open-ended qualitative interview guides were developed (Supplemental Material 1) for informants at each government health system level (national, regional, and district). Study staff gathered names for shortlists of possible key informants at each level in consultation with PMI Impact Malaria country office iCCM technical advisors. These shortlists were refined to target two to three informants per level in each country based on the following criteria: experience and knowledge of CHW programs in their country, access to an Internet connection, and comfort in either conversational English or French.

Study staff contacted key informants via e-mail to inform them of the study and share the study information sheet. After confirmation of interest, each key informant was sent a preinterview request for information and an informed consent form to be reviewed and signed ahead of their interview. Three attempts were made to contact and schedule interviews with each identified informant. Interviews were conducted using videoconferencing and lasted approximately 45 to 60 minutes. Four study members (J. A., A. N., J. M., and A. C.) conducted key informant interviews in French or English. The interviewers administered the interview guide and took notes. All interviews were recorded and were then reviewed to extract relevant information and to supplement interview notes.

### Desk review.

The study team also conducted a desk review to obtain information on the implementation and monitoring of each country’s CHW program, establish a framework for analysis, and identify approaches used for improving the quality of community-based fever management in each country. Relevant documents and materials (listed in Supplemental Material 2) were identified through conversations with key informants, including Ministry of Health (MOH), NMP, and PMI Impact Malaria country staff. Documents and materials included national strategic plans, guidelines, reports, and presentations. The Madagascar, Malawi, and Mali malaria digital community health assessment reports developed by Digital Square (Table 1) (an initiative funded by the United States Agency for International Development [USAID], the Bill & Melinda Gates Foundation, and a consortium of other partners) were also reviewed.^18^^,^^25^^–^^27^

**Table 1:** Overview of community and digital health strategies in study countries

Strategy	Madagascar	Malawi	Mali
National digital health strategy in place	X	X	X
National digital health strategy in place and includes funding	–	X	X
National digital health and community health strategies aligned and support each other	–	X	X
At least one digital tool in use to assess and monitor community health worker quality-of-care level	X	X	X

### Data analysis and validation.

Desk review and interview data from each country were aggregated into one Microsoft Word document per country. The study team convened to review the aggregated data to ensure the information captured was comprehensive, and to identify common themes across the three countries for analysis. The team then held a series of validation meetings with the PMI Impact Malaria Madagascar, Malawi, and Mali country teams to review preliminary findings from each country.

## RESULTS

Of 26 key informants identified, the study team interviewed a total of 10: four from Madagascar (three at the national level and one at the regional level), three from Malawi (one at the national level and two at the district level); and three from Mali (two at the national level and one at the regional level). Seven identified key informants did not respond to an interview request, two declined the invitation to interview, and seven had scheduling conflicts and could not participate. Study results are presented in two parts. We first provide an overview of each country’s community health landscape and QI approaches, then summarize and compare the approaches and their implementation using the four main themes derived from the interviews: training, supervision, coaching/mentoring, and review meetings.

### Country community health strategies and quality improvement approaches.

#### Madagascar.

In Madagascar, QI of fever management at the community level is integrated within national policies and guidelines on community health, with oversight from the Directorate of Primary Care (*Guide Harmonisé de Mise en Œuvre du Program de Santé Communautaire à Madagascar*).^28^ The national community health strategy includes approaches for QI of community health activities, which include control and surveillance of communicable diseases. However, the strategy does not align with the country’s national digital health strategy as it does not discuss any activities related to digital health apart from mentioning a strategic priority of integrating community health data within national data systems. Neither strategy includes plans for the use of digital tools for community health. Madagascar’s mobile connectivity coverage is only 65%, often with very low bandwidth, and just 5% of rural areas have electricity.^26^

Multiple informants explained that, according to the national strategy, community health activities are conducted by volunteer CHWs who are chosen by their communities. Oversight of a community’s health activities, as described in Madagascar’s *Harmonized Community Health Program Implementation Guide^28^* and confirmed by key informants, is provided by *comités de santé* (health committees), which are led administratively by village chiefs and include health facility in-charges, other community groups, and the two CHWs that serve it. The CHWs are managed by the in-charge of their associated health facility. In general, each community site is supposed to serve approximately 1,500 inhabitants. Each site must be either ≥5 km from a health facility or in a particularly inaccessible area. Actual site catchment area size and location could not be confirmed through this study.

Key informants confirmed that community-based QI approaches implemented in Madagascar include training of health staff and CHWs on iCCM, supervision of CHWs, coaching to provide regions and districts with support for community-based activities, and regular (most often monthly or quarterly) meetings between CHWs and their supervising facilities to review community health data and activity implementation. When asked about the contribution of CHWs, one respondent highlighted the critical importance of CHW services:

*Lives are saved with the [CHWs]. We must continue in this direction because for some regions, people are [primarily] consulted at the level of the [community]. (MOH staff member, Madagascar)*

However, one informant noted that financial constraints have left districts in 5 of 23 regions without any support for CHWs or community health activities. In the remaining 18 regions, institutionalization and scale-up of QI efforts have been hampered by constrained resources, such as the lack of dedicated staff to oversee community activities and a disjointed data reporting system. One respondent explained that although a health management information system (HMIS) database exists specifically for community activities, data from paper forms used by CHWs are not regularly digitized or linked to the national HMIS. Furthermore, the community- and facility-level HMIS databases are not linked and must be accessed separately. In some regions, digital data collection by CHWs is being piloted with support from the USAID using the CommCare application v2.53.1 (Dimagi, Inc., Cambridge, MA).

#### Malawi.

In 2017, the Malawi MOH launched its first national community health strategy with the aim of harmonizing community-level health service structures and delivery, building capacity for community health service delivery, and integrating community health commodity management within the national supply chain.^29^ The strategy calls for a strengthened feedback loop between CHWs and their communities, and establishes a hierarchy within the community health system whereby community-level supervisors oversee CHW activities. The strategy includes details on the use of digital tools for community-level data collection and is well aligned with the country’s digital health strategy.

The role of CHWs is institutionalized within the Malawi health system through the provision of a regular wage and supervision. The community health strategy describes the different cadres of CHWs operating nationwide: health surveillance assistants (HSAs), senior HSAs (who supervise HSAs), community health nurses, and community midwife assistants. Health surveillance assistants provide health services such as iCCM from their local health posts. These health posts are intended to serve the 16 million people living in rural areas, including more than 4 million people living in hard-to-reach areas >8 km away from a health facility.

To improve the quality of iCCM service delivery by CHWs after their initial training, all Malawian informants noted that the district health office coordinates supportive supervision, mentorship, stock supply monitoring, review meetings, and refresher training. However, although national policy states that QI activities should be driven by need, one respondent cited that not all QI activities are planned this way:

*Sometimes, activities are not need driven. [We] have to align activities with partners through district planning committees. Sometimes, partner needs are different from what the district needs. (District staff member, Malawi)*

All key informants corroborated the community health strategy by explaining that, in practice, supportive supervision visits are led by the MOH and local district teams that observe CHWs working in their communities and provide feedback and support to improve performance and fill competency gaps. These visits combine supervision, coaching, and action planning. Community health workers also travel quarterly to a local clinic, where they are presented with fever cases to assess and treat in the presence of a facility health provider trained in CHW mentorship. After supportive supervision and mentorship are provided, a review is completed to monitor whether CHWs are restocking and reordering supplies to meet inventory requirements. Last, review meetings are held during which several CHWs from the area gather with a supervisor on a quarterly basis to share lessons learned and to understand gaps more fully in malaria case management at the community level through an exchange of experiences. Community health workers are encouraged to engage with village health committees to analyze QI data, identify problems, and develop ideas for improvement.

One key informant noted that the MOH has developed and rolled out a national community-based health information system to track all community health indicators and provide more insight into CHW performance, allowing for the development of tailored QI interventions. However, another informant noted that the community health strategy’s target of 75% of CHWs collecting data electronically has not yet been met, possibly as a result of low mobile connectivity coverage (18%) and the fact that just 10% of rural areas have electricity.^27^

#### Mali.

According to one respondent, in 2010, Mali launched a national community health strategy that aimed to reach underserved populations through the establishment of CHW health-care delivery sites within hard-to-reach villages. The strategy includes a performance-monitoring framework with well-defined indicators (listed in Supplemental Material 3).^30^ One informant highlighted the importance of using these data to evaluate the performance of QI approaches:

*[We] should always try to evaluate the strategies being implemented. We need evidence that it works before moving forward with it. (MOH staff member, Mali)*

A CHW site covers, both in policy and practice, approximately 700 inhabitants within 3 km of a CSCOM in southern regions and 100 to 500 inhabitants within 25 km of a CSCOM in the less densely populated northern regions (Mali’s *Plan Stratégique National des Soins Essentiels dans la Communauté 2021–2025*).^30^ In addition, CHW sites provide care to populations outside the catchment area of CSCOMs, which are a grouping of villages and/or neighborhoods around a health facility. Two respondents shared that administrative management of CSCOMs is provided by a community health association, and technical oversight is provided by a technical health director recruited and trained on CHW work and supervision. One informant noted that, in 2022, the Malian government formalized the status of CHWs within the health system with a guaranteed salary.

All Malian respondents explained that CHW training is followed by regular CHW supervision visits, which allow for the collection of monthly CHW activity reports and provide insight on the quality of community-based service delivery and data collection. Mali’s CSCOMs also provide mentorship to CHW supervisors, and villages organize regular community steering committee meetings. One informant noted that much of the focus for digital health has been to strengthen the national HMIS and improve monthly reporting by health facilities, including the reporting of community-based services. Data collected by CHWs are entered into the HMIS at the facility level and can be disaggregated from facility-based data. Notable challenges to HMIS reporting cited by all respondents include low Internet bandwidth and unavailability of electricity in rural areas (15%) compared with urban areas (91%).^25^

### Quality improvement approaches and tools.

#### Training.

Approaches to CHW training vary across the three countries (Table 2). Madagascar’s community health strategy includes both theoretical and practical preservice training for new CHWs, but it does not specify the duration of the training. Malawi’s community health strategy mandates 6 weeks of training for all new CHWs. Key informants confirmed that this policy is indeed implemented according to the policy. Community health workers train with CHW supervisors and health providers by studying cases of sick individuals and their care. In Mali, an informant explained that newly hired CHWs receive 21 days of preservice training from CSCOMs, including classroom training and field trip visits to health facilities and/or communities for practical training, in line with the national policy. Another informant shared that Mali’s 3-2-1 telephone service provides toll-free, on-demand access to local language contents of the Mali CHW training modules. The service has been appreciated by users and, anecdotally, helps improve CHW performance, although evidence supporting this claim has not yet been documented. Multiple respondents from Madagascar and Mali cited the difficulty in training all the CHWs in the country because of limited resources, most of which are provided by donors with content or geography-specific priorities. To address this shortage in Madagascar, multiple respondents explained that new CHWs receive 7 days of training at the end of cascaded training of facility workers, who then train CHWs. One key informant noted that Madagascar also prioritizes training for CHWs from villages reporting more malaria cases and those from malaria elimination districts. Another explained that the smartphone-based CommCare app is being used in the 19 regions of Madagascar with support from the USAID to assist CHWs in data collection, reporting, management of notifiable diseases (including malaria), and medication supply management. Community health workers receive 5 to 6 days of training on smartphone use, followed by specific training on data collection using CommCare.

**Table 2:** Overview of CHW training policy and implementation in study countries

Element	Madagascar	Malawi	Mali
Trained individuals	Community health volunteers	Health surveillance assistants	CHWs
Geographic scope	18 of 23 regions	National	National
Trainers	Health facility in-charges	CHW supervisors and health providers	CSCOM technical staff
Training format and content per community health strategy	Training materials should be adapted to the package of interventions provided in the area A classroom training phase is to be followed by a practical phase in the community Training modalities include singing, sketches, case studies, face-to-face learning and e-learning, feedback sessions, before-and-after tests, and self-evaluations	Refresher training (both classroom and practical) include Assessing sick children Identifying signs and symptoms of common illnesses and severe malaria Identifying and administering proper treatment to sick children Counseling caregivers of sick children Conducting follow-up with caregivers Completing village registers and reporting forms	Guides, training manuals, and iCCM management and reporting tools are used during training Training modalities include presentations, case studies and exercises, plenary discussions, field visits, brainstorming, commentary, discussions, and demonstrations
Target duration per community health strategy	One-day TOT that cascades to 1-day CHW classroom training	Initial CHW training is 6 weeks; refresher training lasts 4 days	Initial CHW training is 21 days
Target frequency per community health strategy	Refresher training is conducted three times a year during CHW supervisions	Refresher training is conducted every 2 years or sooner if clinical protocols change	The first refresher training session is conducted 3 months after the initial training. Refresher training is then provided as needed or after a maximum 5 years
Training locations	Health facilities	Training centers	CSCOM facilities

CHW = community health worker; CSCOM = Center de Santé Communautaire (community health center); iCCM = integrated community case management; TOT = training of trainers.

Multiple respondents explained that refresher training for CHWs is conducted in all three countries with varying frequency. A key informant in Malawi recalled a successful refresher training during which it was identified that some CHWs were giving the first dose of oral artemisinin-based combination therapy rather than rectal artesunate (RAS) for prereferral treatment of severe malaria. The informant reported that refresher training increased knowledge and confidence among CHWs to administer RAS, which led to documented increases in its use and anecdotal improvements in case referral for severe malaria:

*Refresher trainings have been used to discuss and address challenges that arise from the community. [Following refresher training, the] confidence of providers was boosted in providing care to sick children at the community level. (District staff member, Malawi)*

#### Supervision.

All three countries have CHW supervision plans documented in their community health strategies (Table 3). Respondents in all three countries noted that the focus and timing of supervision visits often depend on donor priorities and commitments as well as on the availability of supervisory staff. As a result, none of the three countries reported having adequate human or financial resources to supervise all their CHWs. In Madagascar, one informant noted that just 35% of CHWs (those in 40 of 114 districts) receive any form of supervision. A Malawian informant explained that if a donor-funded partner organization is not present within a district to support supervision, the district must wait for the MOH to organize resources for supervision. Most respondents mentioned the importance of CHW supervision after training and the need for external funding to support this:

*The supervisions allow for close monitoring of the case management and how the [CHW] is entering data in their registers. [Supervisors] can check for accuracy and make corrections in real time. (Regional staff member, Madagascar)*

*Specific activities may depend on partners and funding. Without partners, it’s hard to maintain regularity of trainings and supervisions. (MOH staff member, Mali)*

**Table 3:** Overview of CHW supervision policy and implementation in study countries

Element	Madagascar	Malawi	Mali
Term used for CHWs	Community health volunteers	Health surveillance assistants	CHWs
CHW supervisors	Health facility in-charges (occasionally, CHWs do peer supervision)	Senior health surveillance assistants	CSCOM-dedicated supervisors
Geographic scope	40 of 114 districts	National	National
Target frequency per community health strategy	Monthly	Quarterly	Monthly
Actual frequency	Quarterly	Quarterly, but only for up to 15% of community health clinics in the supervisor’s assigned area	Quarterly
Supervision tool	CHW supervision checklist (paper and digital)	CHW supervision checklist (paper)	CHW supervision checklist (digital); Digitalisation de la Santé Communautaire au Mali dashboard (digital forthcoming) (Medic Mobile, Bamako, Mali)
Supervision data reporting and use	Development and review of CHW action plans, manual data entry and analysis in Excel	Development and review of CHW action plans, manual data entry and analysis in Excel	Development and review of CHW action plans, digital data entry and analysis in Excel, monthly upload to health management information system (forthcoming)

CHW = community health worker; CSCOM = Center de Santé Communautaire (community health center).

If CHWs are supervised, they often do not receive supervisory visits at the frequency dictated in the national community health strategy because of funding and human resource gaps. As a result, respondents said that CHW supervision in Madagascar and Mali is done quarterly instead of monthly. In Malawi, one informant noted that supervisors can often only conduct supervisory visits quarterly for one to two of the approximately 14 community health clinics in their assigned area.

Informants explained that in Malawi and Mali, supervision is done by dedicated supervisors with no competing professional tasks beyond medicine distribution, whereas in Madagascar, supervisors are health facility in-charges. These in-charges either supervise their CHWs during meetings at the facility or during visits to the communities in which the CHWs work. Obtaining approval for supervisory visits is time-consuming and depends on the motivation of the health facility in-charges, who are frequently the only staff at the facility and may find it difficult to leave their post to supervise CHWs in the community. One respondent explained that this has led to an arrangement in a few districts whereby the health facility in-charge identifies and oversees a well-performing CHW to conduct peer supervision:

*If the [health facility in-charge] is the only person in the [health facility], it is challenging for them to leave to conduct the supervisions. Their travel [for the supervisions] may also be logistically challenging. Due to lack of funds, not all the [CHWs] are able to receive a supervision visit. (MOH staff member, Madagascar)*

One respondent from Malawi explained that the lack of support for supervision staff (senior HSAs) transportation greatly hampers the implementation of the approach because commuting to the CHW’s community can be expensive:

*There are issues in relation to where the [supervisor] stays and where the clinic is. Some [supervisors] do not have a house in the catchment area and there is a need to commute to the clinic. This can be expensive for them, especially if they do not have a bike. (MOH staff member, Malawi)*

In Mali, one respondent explained that the first CHW supervisory visit is supposed to be completed within 30 days of the CHW starting work, followed by monthly supervisions of 12 to 18 CHWs by CSCOM-dedicated supervisors. Mali’s supervisors are also responsible for on-the-job training and disbursement of supplies and equipment to CHWs. National and regional teams in Mali conduct biannual supervisions, and district and health facility teams conduct quarterly supervisions.

*[The supervision at the national and regional levels in Mali] are to evaluate not only the CHW, but also to try and understand the impact of supervision on the quality of the CHW’s work. (MOH staff member, Mali)*

In all three countries, key informants described how supervisors use MOH-validated, observation-based checklists to assess CHW performance, including questions to evaluate CHW competency in iCCM and malaria service delivery during a sick child consultation and referral, and reporting through a review of the CHW’s consultation forms and registers. Availability of commodities is also monitored through the checklists. Respondents in Madagascar and Malawi explained that paper-based versions of their CHW checklists (see Supplemental Materials 4 and 5, respectively) are used, except in three districts of Madagascar supported by PMI Impact Malaria, where the checklist has been digitized. The project supports the NMP in the oversight of the tool and its data, which are downloaded and analyzed in Excel. In Mali, a new application is currently being digitized to allow CHWs to collect data electronically, and for supervisors to assign tasks to the CHW, and analyze and visualize activity and performance data. Supervisors will also be able to monitor a CHW’s longitudinal follow-up of patients by accessing these data disaggregated to the level of the patient, which would allow CHWs and their supervisors to identify and track areas for improvement immediately. Community health workers will be expected to send data from the tool to the HMIS server using the cellular network at least twice per month. Community health worker supervisors are currently being trained on the tool and CHW training will follow.

Respondents from all three countries described how the supervisor and the CHW review the supervisory results before developing and agreeing on action plans that will be followed up by the supervisor during subsequent visits. These action plans are considered important to improving CHW performance as well as to sustain gains through community engagement in the implementation of the QI approach. A respondent from Madagascar noted that action plans are also posted on the walls of the CHW’s associated health facility:

*Regular supervisions [and] action plans developed after the supervision help reinforce the sustainability of the QI. (Regional staff member, Mali)*

*The sustainability of an approach that is essentially conducted at the community level, the results must be shared with the community on a regular basis so that the community can understand the approach, express itself, and play its role. (Regional staff member, Mali)*

*There has been community mobilization as a result of supportive supervision. Each clinic has a committee of 10 members that help in mobilizing people and acts as a bridge between community and chiefs. [They] can discuss [CHW] issues as well, such as building a house for the [CHW] in the catchment area. (MOH staff member, Malawi)*

Supplemental Materials 6 and 7 show examples from Madagascar and Mali of how indicators calculated from CHW supervisory data are analyzed routinely and presented.

According to the Malawi MOH, CHW supervisions may have contributed to the improvements recorded in malaria case management and in the reduction of under-five all-cause mortality.^31^

#### Coaching/mentoring.

Respondents explained that Malawi and Mali organize mentoring for their CHWs and CHW supervisors, respectively. In Malawi, two respondents noted that relatively low-performing CHWs receive mentorship based on supervisory results. These CHWs travel to their local health facility, where they assess and treat three to five fever cases in the presence of a facility health provider who has been trained as a mentor:

*This [quality of care] assessment approach looks at the skills of CHWs at the lower level. … When that is administered, it looks at combining supervision with coaching right at that time. (District staff member, Malawi)*

As with supervision, there are often limited funds for CHW travel to health facilities. Mentor attrition is another challenge commonly cited by informants in Malawi, meaning that practice does not always align with mentorship policy:

*[For staff] to provide care at the health facility, but also have time to provide community case management mentorship, is a challenge [because] there are only one or two individuals who are doing mentorship to more than 10 [CHWs]. (District staff member, Malawi)*

In Mali, per the national guidelines, the CHW supervisor is mentored and coached monthly by the CSCOM technical director through direct observation of supervisory visits and the use of a monitoring checklist. The technical director and supervisor discuss feedback and areas for improvement. In Madagascar, two respondents explained that a nutrition-focused project assigns coaches to motivate and encourage regions and districts within their project scope to coordinate the mobilization and sharing of malaria information and resources, and improve their malaria situations through proper prevention techniques and care seeking.

#### Review meetings.

Key informants corroborated national community health strategy documentation describing how all three countries organize meetings to review community health activities, although each country assembles a different group of stakeholders and reviews different aspects of the work (Table 4). In Madagascar, respondents explained that CHWs are supposed to travel to their associated health facility for monthly meetings with the in-charges to review CHW registers, reports, and forms, and to conduct quality control of their data. However, lack of funding and resources for printing and transport means that CHWs may not have all the forms they need or may not be able to travel to the facility for each month’s meeting. One respondent noted that, in some instances, CHWs request money or nonfinancial incentives before they agree to send their data and attend the meeting:

*[CHWs] are only volunteers, but they are now asking for incentives. … There are times where they go on strike—for example, during [insecticide-treated net] distribution campaigns. For example, they don’t send back the data and are requesting money. (MOH staff member, Madagascar)*

**Table 4:** Overview of community health review meeting policy and implementation in study countries

Element	Madagascar	Malawi	Mali
Meeting participants	Community health volunteers, health facility in-charges	Health surveillance assistants; CHW supervisors	CHWs, members of the pilot committee
Target frequency per community health strategy	Monthly	Quarterly	National level: biannually CHW sites: monthly
Objectives	CHW register, reports, and forms review; data quality control; collection of commodities	CHW data review, problem solving	Discussion of the implementation of community approaches, exchange of solutions to problems

CHW = community health worker.

Groups of CHWs in Malawi meet with a supervisor at a set location to review their data and share best practices and solutions to challenges, according to one respondent. These cluster review meetings are intended to occur every quarter, although they are often held less frequently because of lack of funding for travel:

*Resource needs [such as fuel, vehicles, and lunch allowances] are a limitation for conducting cluster review meetings. The key [solution to resource challenges] is integration [of] any of these pieces into other QI tools. (District staff member, Malawi)*

In Mali, all community-based interventions are coordinated by the General Directorate of Health and Public Hygiene through steering committees that exist at all levels of the health system. At the community level, each CHW site has its own committee, which is presided over by the site’s village chief and includes CHWs, religious leaders, traditional providers, women’s and youth associations, and other groups. One informant explained that its role is to increase community awareness and mobilization around the health activities provided by the CHWs:

*The [committees] are key to sustaining the community-level work [by helping to] coordinate activities at all levels, and create transparency and accountability for the implementation of the community-level activities. (MOH staff member, Mali)*

These steering committees are supposed to meet monthly to review implementation of community health activities, propose solutions to challenges encountered, and plan the following month’s activities. However, two respondents noted that they often do not meet as frequently as planned, and the Directorate is disseminating additional guidance to committees to improve their planning and management.

## DISCUSSION

These country case studies provide historical and contextual information and describe approaches currently used in Madagascar, Malawi, and Mali to improve the quality of community-based fever management. Despite the diversity in settings, health system structures, and the level of institutionalization of community health, all three countries have well-defined community health strategies that include QI approaches and tools covering CHW and supervisor training, supervision, coaching/mentoring, and review meetings. Some of the approaches and tools we examined are not integrated within the countries’ community health strategies. In addition, some approaches are for QI of iCCM activities, whereas others focus specifically on QI of malaria case management.

All three countries included in our study face a crucial challenge in implementing their community-based QI approaches: the persistent lack of resources, particularly human and financial. As a result, none of the countries can extend their full QI package at the desired frequency to all CHWs.

Our study highlights key facets of this challenge and shares possible solutions. First, there are not enough QI staff to implement the approaches, and those who are available are often constrained by a lack of time or funds to complete the work. Health facility staff are often asked to balance running their facilities (where they may be the only health provider) with implementing QI activities, and may see no other option than to prioritize their clinical practice. Even if QI staff do not have competing priorities, they are often unable to visit CHWs at the desired frequency as a result of the lack of transportation funds. Conversely, the CHWs they supervise may not get compensated, incentivized, or reimbursed for travel for training, supervision, mentoring/coaching, or review meetings.

Although country community health strategies in all three countries integrate multiple health areas, in practice, QI activities are often focused on specific health areas that receive vertical donor funding. In some instances, this funding is also tied to specific geographic areas, limiting coverage of QI to only a portion of CHWs in the country. For example, in Madagascar, a nutrition-focused project has dedicated supervisors for the CHWs whose work they fund. The project holds monthly review meetings with these CHWs to ensure sustainability of the QI approaches used. However, CHWs who are not funded by the project are not included in the meetings.

Our study identifies some solutions that countries have used to strengthen the implementation of community-based QI activities, especially those facing resource challenges. As is already the case in the three countries, coupling and streamlining QI activities—such as combining training and supervision or training and group problem solving—can mutually strengthen and sustain them^32^^,^^33^ while minimizing their implementation costs.^32^^,^^34^ Integrated QI approaches at the health facility level, such as OTSS, have also been shown to contribute to improved competency in fever management.^35^ Efficiencies in QI implementation through cascade training for multiple levels of a health system are considered a cost-effective and efficient approach to reach all targeted participants in Madagascar. Having more trained QI staff can strengthen supervisory quality, which has been shown to contribute to stronger CHW performance.^36^

Our study also highlighted that communities and districts may be best placed to implement, monitor, and address challenges to community-based QI approaches. However, they require the human and financial resources as well as the mechanisms to facilitate engagement with the health system for timely and effective QI implementation (such as supply chain management and approvals for logistics or release of funds) to avoid placing further stress on overburdened staff. This arrangement would capitalize on the strong ties and mutual support that CHWs in all three countries have with the communities they serve. Community support for CHW activities has been shown to have a positive effect on improving CHW performance,^32^ and the WHO encourages community engagement and feedback on the quality of CHW service delivery to complement supervisory efforts.^4^

Digitizing tools used for the collection, reporting, and analysis of CHW data, as well as CHW supervisory tools, can help mitigate recurring costs associated with printing and transport of paper-based tools. It can also help supervisors with automated calculation of performance indicators, real-time availability of data, user-friendly scoring of performance to tailor feedback, and easy access to previous supervision results, which allows them to monitor progress over time and identify persistent gaps. Studies have demonstrated that digitizing tools can facilitate access, interpretation, and action based on complete and timely data collected at the community level.^37^^–^^40^ In Malawi and Mali, where the national digital health strategy is well aligned with the national community health strategy, tools used for the collection, reporting, and analysis of community data are digitized and integrated within an HMIS. Data can also be disaggregated by level, thereby allowing action plans to be tailored to each community. In countries where CHW data are not integrated into the national HMIS, and where use of digital tools at the CHW level is not feasible, disaggregation between facility- and community-based data may be achieved through the addition of a CHW-specific section to the monthly facility health services report, which was noted by informants as a best practice in Mali.

One key informant emphasized that community-based QI activities should be monitored through performance data on a trial basis before being scaled up. This type of routine monitoring can be made more feasible, effective, and efficient if community health programs identify a small number of high-value performance indicators that can be measured and interpreted easily, and are useful to global and national stakeholders as well as health providers.^41^

Our study has some limitations. Study timelines and nonresponses by potential key informants limited data collection efforts. As a result of the smaller number of respondents than initially planned, some caution is warranted in generalizing these findings. Overall, though, there was a balanced mix of national and subnational health authorities interviewed for this study. Although efforts were made to conduct a comprehensive desk review, the study team may not have obtained all the relevant documents and materials for this review.

Quality improvement activities are critical to ensuring febrile patients receive appropriate care at the community level. Strong, integrated, community-based QI strategies and programs must be pragmatic, feasible, and resource conscious to achieve their goals. The overarching goal should be an integrated, country-led QI program with priorities that are driven by country needs to ensure consistent CHW support and sustainability. Through coordination and pragmatic planning, a variety of adaptive QI approaches can support the implementation of successful community programming for fever management and beyond.

## Supplemental Materials

10.4269/ajtmh.23-0488 Supplemental Materials
