# Supplementary material for: Experiences in Improving the Quality of Community-Based Fever Management from Three Malaria-Endemic African Countries
Source: Am J Trop Med Hyg. 2024 Jan 9;110(3 Suppl):66–75. doi: 10.4269/ajtmh.23-0488 (PMC10919238; doi:10.4269/ajtmh.23-0488)
Supplement: Supplemental Materials [file tpmd230488.SD1.pdf]

Supplementary Materials

Ahmed, J. et al. Experiences in improving the quality of community-based fever management from 3 malaria-endemic African countries.

S1: Key Informant Interview Guide	Page 2
S2: Documents and Materials Included in the Study Desk Review	Page 8
S3: Mali Community Health Strategy Indicators	Page 9
S4: Madagascar CHV Supervision Checklist	Page 13
S5: Malawi CHW Supervision Checklist	Page 18
S6: Madagascar CHV Supervision Checklist Data Visualization	Page 20
S7: Mali CHW Supervision Checklist Data Visualization	Page 21

<u>S1: Key informant interview guide.</u>
Experiences in improving the quality of community-based fever management from 3 malaria-endemic African countries

Qualitative Interview Guide

Pre-Interview questions (for all levels):
Before conducting interviews, let's use these questions to acquire information ahead of time and we can use the answers to guide the interview and we can validate the information during that time.
Before interview, copy and paste in answers for reference during interview.

- Which quality improvement approaches are being used in your country to improve community-based fever management? Have there been policy documents and guidelines produced, trainings conducted, peer-to-peer learning, rally posts, supervision, internships, etc.?
- Where is each approach being used and since which year?
- How is each approach implemented? (basic bullet points are okay)
- Which tools or strategies are currently being used to generate evidence from each approach described above? Is there an inventory checklist, competency-based iCCM checklist, RDT performance checklist, HMIS, etc.?
- How are these tools or strategies implemented? (bullet points are okay)
- Can you send us examples that demonstrate how the evidence generated by these tools or strategies is presented (e.g., graphs/tables/text/maps/etc. in reports, presentations, guidance documents, summaries, websites, or other sources)?

**Interview date**	
**Respondent name**	
**Position**	
**Organization**	
**Respondent category**	National, regional, or district
**Additional information about the respondent**	
**Interviewer name**	
**Start time**	
**End time**	

National level interview questions:

- Introductions and explanation of the interview
  - Hello, my name is __________, and I'm part of the PMI Impact Malaria project. We're here to chat about how you have been implementing quality improvement approaches for community-based fever case management. PMI Impact Malaria is looking to consolidate learnings across countries in sub-Saharan Africa to share lessons with the global community from programs currently implementing quality improvement for community-based fever case management.

- Tell me a little about your work with quality improvement in general as well as with CHWs. We generally define quality improvement as a series of processes used to continuously maintain and improve quality. In the case of iCCM, these processes may be training, the regular supply of CHWs with materials and commodities, incentives and supportive supervision as well as well-defined indicators to monitor results and address gaps and take actions for continuous improvement.
- Tell me about your work with quality improvement specifically regarding community fever case management.
- In your written response, you mention that you are using the following approaches for quality improvement to improve community-based fever management in [location] since [year]. Are there any other approaches?
    - What successes did each approach's implementation present?
    - What challenges did each approach's implementation present?
        - *Be sure each approach is covered (both successes and challenges)*
        - How have the challenges been addressed, if at all?
    - What are the operational strengths of each approach, if any? *(The interviewer can guide through each approach rather than relying on the interviewee to go through the exhaustive list)*
    - What are the operational weaknesses of each approach, if any?
    - Are there plans to further strengthen any of these approaches? In which ways?
    - Has the approach been scaled up?
        - IF YES: Where was it first implemented and where has the approach been scaled up to?
        - IF YES: Was it easy or difficult to scale up the approach? (Probe for more information on why it was easy or difficult)
        - IF NO: Are there plans to scale up any of the approaches? How?
    - What are some ways to encourage sustainability of these approaches?
        - Are these methods of sustainability currently being implemented?
        - If not, what are the next steps to implement methods of sustainability? Are there any barriers in moving forward to implement methods of sustainability?
- Are there any resources that you can share with us on quality improvement of community-based fever case management from your country?
- In your written response, you mention that you are using the following tools or strategies to generate evidence from each approach. Are there any additional tools or strategies?
    - [Strategy/tool] was implemented [process]. Which successes did you find this presented?
    - [Strategy/tool] was implemented [process]. Which challenges did you find this presented?
        - Interviewer can do this (both challenge and success) for each strategy/tool
    - What are the operational strengths of each strategy/tool, if any? *(The interviewer can guide through each strategy/tool rather than relying on the interviewee to go through the exhaustive list)*
    - What are the operational weaknesses of each strategy/tool, if any?
    - Are there plans to further strengthen any of these strategies/tool? In which ways?

- Are there plans to scale up any of the strategies/tool? How?
    - IF YES: Where was it first implemented and where has the approach been scaled up to?
    - IF YES: Was it easy or difficult to scale up the approach? (Probe for more information on why it was easy or difficult)
    - IF NO: Are there plans to scale up any of the approaches? How?
- Were there previous attempts to produce and utilize tools and/or strategies related to quality improvement of community-based fever management?
    - If yes, why are they no longer used?
    - Were there any important lessons learned from those attempts?

If not answered from the previous questions, here are additional questions:

- Do you have other ideas or thoughts about how best to approach quality improvement for community service delivery?
- How do CHWs maintain their motivation?
- Is there anything else you feel would be important for me to know?
- What questions do you have for me?

Regional level interview questions:

- Introductions and explanation of the interview
    - Hello, my name is __________, and I'm part of the PMI Impact Malaria project. We're here to chat about how you have been implementing quality improvement approaches for community-based fever case management. PMI Impact Malaria is looking to consolidate learnings across countries in sub-Saharan Africa to share lessons with the global community from programs currently implementing quality improvement for community-based fever case management.
- Tell me a little about your work with quality improvement and community fever case management in your region. We generally define quality improvement as a series of processes used to continuously maintain and improve quality. In the case of iCCM, these processes may be training, the regular supply of CHWs with materials and commodities, incentives and supportive supervision as well as well-defined indicators to monitor results and address gaps and take actions for continuous improvement.
    - Do you feel this approach is appropriate for your region?
    - Has this approach been accepted by personnel in your area?
- In your written response, you mention that you are using the following approaches for quality improvement to improve community-based fever management in [location] since [year]. Are there any other approaches?
    - What successes did each approach's implementation present?
    - What challenges did each approach's implementation present?
        - *Be sure each approach is covered (both successes and challenges)*
    - How have the challenges been addressed, if at all?
    - What are the operational strengths of each approach, if any? *(The interviewer can guide through each approach rather than relying on the interviewee to go through the exhaustive list)*
    - What are the operational weaknesses of each approach, if any?
    - Are there plans to further strengthen any of these approaches? In which ways?
    - Has your region adapted these approaches? How?
    - Are you the only region that performs these approaches?
        - If so, why?

- What are some ways to encourage sustainability of these approaches?
  - Are these methods of sustainability currently being implemented?
  - If not, what are the next steps to implement methods of sustainability? Are there any barriers in moving forward to implement methods of sustainability?
- How do you feel these approaches are sustainable? What does sustainability look like to you? Are there barriers from the national level that inhibit sustainability? *For example national policy, approval processes, etc.*
  - If not, how do you make them sustainable?
  - If so, how do you maintain sustainability?

- Are regions able to produce their own quality improvement strategies, tools, or resources such as job aids? If no, do all resources need to come from the national level?
  - If yes, are there any resources that you can share with us on this topic from your region?
- In your written response, you mention that you are using the following tools or strategies to generate evidence from each approach. Are there any additional tools or strategies?
  - [Strategy/tool] was implemented [process]. Which successes and challenges did you find this presented? *(Interviewer can do this for each strategy/tool)*
  - What are the operational strengths of each strategy/tool, if any? *(The interviewer can guide through each strategy/tool rather than relying on the interviewee to go through the exhaustive list)*
  - What are the operational weaknesses of each strategy/tool, if any?
  - Are there plans to further strengthen any of these strategies/tools? In which ways? What are the inhibitors to doing this?
  - Were there previous attempts to produce and utilize tools and/or strategies related to quality improvement of community-based fever management in your region?
    - If yes, why are they no longer used?
    - Were there any important lessons learned from those attempts?
  - Do you know which districts are performing these activities?
    - Is it possible for a district to strengthen approaches themselves or must it come from the regional or national level?

If not answered from the previous questions, here are additional questions:

- Do you have other ideas or thoughts about how best to approach quality improvement for community service delivery?
- How do CHWs maintain their motivation?
- Is there anything else you feel would be important for me to know?
- What questions do you have for me?

District level interview questions:

- Introductions and explanation of the interview
  - Hello, my name is __________, and I'm part of the PMI Impact Malaria project. We're here to chat about how you have been implementing quality improvement approaches for community-based fever case management. PMI Impact Malaria is looking to consolidate learnings across countries in sub-Saharan Africa to share lessons with the global community from programs currently implementing quality improvement for community-based fever case management.

- Tell me a little about your work with quality improvement and community fever case management in your district. We generally define quality improvement as a series of processes used to continuously maintain and improve quality. In the case of iCCM, these processes may be training, the regular supply of CHWs with materials and commodities, incentives and supportive supervision as well as well-defined indicators to monitor results and address gaps and take actions for continuous improvement.
    - Do you feel this approach is appropriate for your district?
    - Has this approach been accepted by personnel in your area?
- In your written response, you mention that you are using the following approaches for quality improvement to improve community-based fever management in [location] since [year]. Are there any other approaches?
    - What successes and challenges did each approach's implementation present? *(Be sure each approach is covered)*
    - How have the challenges been addressed, if at all?
    - What are the operational strengths of each approach, if any? *(The interviewer can guide through each approach rather than relying on the interviewee to go through the exhaustive list)*
    - What are the operational weaknesses of each approach, if any?
    - Are there plans to further strengthen any of these approaches? In which ways?
    - Are you the only district that performs these approaches?
        - If so, why?
        - Is it possible for a district to strengthen approaches themselves or must it come from the regional or national level?
    - What are some ways to encourage sustainability of these approaches?
        - Are these methods of sustainability currently being implemented?
        - If not, what are the next steps to implement methods of sustainability? Are there any barriers in moving forward to implement methods of sustainability?
    - Are there barriers from the regional or national level that inhibit sustainability? *For example national policy, approval processes, etc.*
        - If not, how do you make them sustainable?
        - If so, how do you maintain sustainability?
- Are districts able to produce their own resources such as job aids? If no, do all resources need to come from the regional or national level?
    - If yes, are there any resources that you can share with us on this topic from your district?
- In your written response, you mention that you are using the following tools or strategies to generate evidence from each approach. Are there any additional tools or strategies?
    - [Strategy/tool] was implemented [process]. Which successes and challenges did you find this presented? *(Interviewer can do this for each strategy/tool)*
    - What are the operational strengths of each strategy/tool, if any? *(The interviewer can guide through each strategy/tool rather than relying on the interviewee to go through the exhaustive list)*
    - What are the operational weaknesses of each strategy/tool, if any?
    - Are there plans to further strengthen any of these strategies/tools? In which ways? What are the inhibitors to doing this?

    - Were there previous attempts to produce and utilize tools and/or strategies related to quality improvement of community-based fever management in your district?
        - If yes, why are they no longer used?
        - Were there any important lessons learned from those attempts?

If not answered from the previous questions, here are additional questions:

- As a district-level individual, you have unique opportunities to hear directly from CHWs related to fever case management and about quality improvement of that work. What have you been hearing from CHWs related to this?
- Do you have other ideas or thoughts about how best to approach quality improvement for community service delivery?
- How do CHWs maintain their motivation?
- Is there anything else you feel would be important for me to know?
- What questions do you have for me?

S2: Documents and Materials Included in the Study Desk Review.

	Documents and materials
**Madagascar**	• Ministère de la Santé Publique. Paquet d'Activités Communautaires Pour Les Agents Communautaires.<br>• Ministère de la Santé Publique. 2022. Guide Harmonisé de Mise en Œuvre du Programme de Santé Communautaire à Madagascar.<br>• Ministère de la Santé Publique. Madagascar Cadre d'Investissement sur la Santé Communautaire 2021 – 2025.<br>• Ministère de la Santé Publique. 2021. Manuel de Supervision Formative des AC ou Agents Communautaires, 2021,<br>• Ministère de la Santé Publique. Plan Stratégique National de Lutte Contre le Paludisme, 2018-2022.<br>• Programme National de Lutte Contre le Paludisme, Ministère de la Santé Publique. 2019. Plan d'Elimination du Paludisme à Madagascar 2019 – 2022. Antananarivo, Madagascar : Programme National de Lutte Contre le Paludisme<br>• Programme National de Lutte Contre le Paludisme. 2022. Surveillance du Paludisme à Madagascar en S38 (19 Au 25 Septembre 2022) SITREP N°38/22.<br>• Programme National de Lutte Contre le Paludisme, Ministère de la Santé Publique. 2020. Bulletin Trimestriel du Paludisme Programme National De Lutte Contre Le Paludisme, Numéro : 16 Février 2022. Antananarivo, Madagascar : Programme National de Lutte Contre le Paludisme<br>• PATH. 2021. Role Of Digital Tools in Fighting Malaria at The Community Level: Madagascar. Seattle: PATH
**Malawi**	• PMI Impact Malaria Malawi. 2021. PMI Impact Malaria Malawi Project Integrated Community Case Management (iCCM) Supportive Supervision November 2021<br>• PMI Impact Malaria Malawi. 2022. PMI Impact Malaria Malawi Project Integrated Community Case Management (iCCM) Supportive Supervision February 2022<br>• PMI Impact Malaria Malawi. 2022. PMI Impact Malaria Malawi Project Integrated Community Case Management (iCCM) Supportive Supervision June 2022<br>• PMI Impact Malaria Malawi. 2022. PMI Impact Malaria Malawi Project Integrated Community Case Management (iCCM) Supportive Supervision September 2022<br>• IMCI Semi-Annual Report, Jan-June 2022. IMCI Sub-TWG Meeting, September 8, 2022.<br>• Government of the Republic of Malawi Ministry of Health and Population. 2020. *Digital Health Strategy 2020-2025*. Malawi: Ministry of Health and Population.<br>• Government of the Republic of Malawi Ministry of Health. 2017. National Community Health Strategy 2017-2022: Integrating health services and engaging communities for the next generation. Malawi: Ministry of Health<br>• PATH. 2021. Role of digital tools in fighting malaria at the community level: Malawi. Seattle: PATH
**Mali**	• Ministère de la Santé et du Développement Social, Direction Générale de la Santé et de l'Hygiène Publique. 2021. Soins Essentiels dans la Communauté – Guide National de Mise en Œuvre *[Essential Care in the Community – National Implementation Guide]*. Mali: Ministère de la Santé et du Développement Social<br>• Ministère de la Santé et du Développement Social, Direction Générale de la Santé et de l'Hygiène Publique, Sous-Direction des Etablissements Sanitaires et de la Règlementation. 2021. Plan Stratégique National des Soins Essentiels dans la Communauté 2021-2025 *[National Strategic Plan of Essential Care in the Community 2021-2025]*. Mali: Ministère de la Santé et du Développement Social<br>• PATH. 2021. Role of digital tools in fighting malaria at the community level: Mali. Seattle: PATH

S3: Excerpt of Mali's Community Health Strategy Indicators.

Performance Indicators of the strategic plan

**Expected results**	**Indicators**
**General objective of the 2021-2025 strategic plan**	
The morbidity and mortality of the populations, in particular those of mothers and children under five, have decreased in Mali by 2025	Infant child mortality is reduced by 2025
	Child mortality is reduced by 2025
	Maternal mortality is reduced by 2025
	Neonatal mortality is reduced by 2025
**Strategic results of the plan**	
RS1: An environment favorable and conducive to the implementation of essential care in the community during the period of 2021-2025 is created	
Implementing essential care in the community evolves within an institutional, legislative and regulatory framework	Number of legislative texts adopted in the context of the implementation of essential care in the community
A financing mechanism for essential care in the community is operational by 2025	% of health budget allocated to financing essential care in the community
The coordination/partnership for the implementation of essential care in the community is strengthened	A coordination/partnership mechanism is functional
The ASACOs and *Collectivites Territoriales* consider essential care in the community in their biannual workplan	Proportion of ASACOs considering community health in micro plans
	Proportion of collectives considering community health in annual workplan and budget
Community engagement in the implementation of community health is strengthened	% of CHWs with a worksite
	% of CHWs benefitting from a motivation other than a salary
RS2: The system for providing care and services for better access of populations to quality service structures in an equitable manner at the community level is strengthened by 2025	
Reinforcing the management of service delivery sites providing essential care in the community in terms of personnel, drugs, inputs, equipment and tools	Rate of essential medicine stockouts
	Rate of input stockouts
	Rate of availability of equipment and tools
The country's coverage of CHW sites is strengthened and meets the establishment standards defined in the SEC 2021-2025 plan	The CHW site coverage rate increases from 23% to 75% from now until the end of 2025
RS3: Demand for better use of health services at community level is increased by 2025	
Communities have taken ownership of the essential community care through the strengthening of integrated communication	Communities participate in community health promotion through the engagement and motivation of CHWs

	Communities organize and support the CHW in the organization of activities
Socio-cultural factors and practices limiting the smooth implementation of community health factors are limited	The demand for and use of community health care and services are increased
RS4: Quality of care and services at the community-level is reinforced by 2025	
All CHWs have adequate equipment	% of CHW sites that have adequate equipment
All community health actors benefit from capacity strengthening from now until 2025	% of the community health actors that have received an orientation on community health
	% of community health actors that are equipped
Community health actors have monthly activity plans that are developed, shared, and implemented	Availability of a developed and shared monthly activity plan
The system of referral and counter-referral between the village and the CSCOM is functional	Number of cases referred from village to the CSCOM through the means of transport put in place
	Proportion of cases referred from the CHW to the CSCOM
	Proportion of children benefitting from pre-referral rectal artesunate
A community health information system is available for the management of community interventions	Availability of a community health information system for the management of community intervention data
The quality of service provision by community health actors is improved through supervision	% of community health actors who carry out activities in alignment with their portfolios
At least 80% of CHW sites are performing well	% of CHWs who carry out activities according to the implementation guide
	Proportion of children under age 5 presenting with uncomplicated diarrhea treated with ORS and Zinc by CHWs
	Proportion of children under age 5 presenting with uncomplicated malaria treated with ACT by CHWs
	Proportion of children under age 5 presenting with pneumonia treated with amoxicillin by CHWs
	Proportion of children under age 5 presenting with moderate acute malnutrition managed with plumpy suppository and enriched flour by CHWs
	Proportion of children under age 5 with uncomplicated severe acute malnutrition managed with plumpynut by CHWs
	Proportion of cases presenting with a danger sign or warning referred by CHWs

	Number of new family planning users
	Proportion of children from age 2 to 5 that received deworming during campaigns
	Proportion of households with a handwashing facility
	Proportion of households with an improved toilet
	Proportion of children from households with access to drinking water
	Proportion of children from 0 to 5 years from households using iodized salt
	Proportion of children from 0 to 28 days who received the 8 visits
	Proportion of children from 0 to 28 days who received umbilical cord care (chlorhexidine)
	Proportion of children from 0 to 6 months exclusively breastfed
	Proportion of children from 1 to 24 months that sleep under an LLIN
	Proportion of children aged 14 weeks who received *Rota 3, PCV13_3, VPO 3, VPI*
	Proportion of children aged 9 months who received *VAR 1, VAA, MenAfricVac*
	Number of suspected tuberculosis cases referred by CHWs
The quality of services offered by community health actors is improved	% of community health actors offering quality services
RS5: The governance, leadership and accountability of all actors involved in the implementation of SEC are strengthened by 2025.	
The capacities of actors at all levels are strengthened in terms of community governance	% of actors who have benefited from capacity building in in community governance
The state provides leadership for all community health activities	All community health interventions/initiatives are authorized, coordinated, and monitored by the state
The collectives, the ASACOs and the communities have taken ownership of the community health strategy	% of communities involved in the implementation and funding of community health
	% of ASACOs and communities participating in the implementation and funding of community health
The actors report on the implementation of community health activities to the community	% of actors regularly reporting on the execution of their role and missions in the implementation of community health activities
RS6: A monitoring and evaluation system of the SEC is set in place and is operational from 2021-2025.	
Community health implementing actors achieve the monitoring and	A performance measurement framework is developed to track

evaluation of community interventions in their respective areas at the periodicity indicated in the plan	community health activities to achieve each year
	% of actors with capacity to implement community health activities with strong planning and monitoring and evaluation
A functional health information system that ensures community data completeness and timeliness	A digitized tool for the management of the CHW's community-based health data and integrating data collected by community-level actors is designed
The monitoring of community interventions is carried out at the *aire de sante* level at the planned periodicity	Number of *aires de sante* that organized monitoring of community interventions at the planned periodicity
Community health actors are informed of the results of studies and evaluations carried out in the context of community health implementation	The results of the studies and evaluations carried out in the context of community health implementation are shared

Extracted and translated from : Ministère de la Santé et du Développement Social., 2021. *Plan Stratégique National des Soins Essentiels dans la Communauté 2021-2025, pg 82-92*. Mali: Ministère de la Santé et du Développement Social

S4: Madagascar CHV Supervision Checklist.

Translated by PMI Impact Malaria from Malagasy to English.

SUPERVISION CHECKLIST FOR THE COMMUNITY HEALTH VOLUNTEERS (CHV)

I. IDENTIFICATION OF THE COMMUNITY SITE

Region	:
District	:
Commune	:
Health Center	:
Village	:
Name of the site	:
First and last name of the CHV	:
Type of CHW (Polyvalent CHV – AC PARN CHV – PCIMEC CHV – Others)	:
Contact of the CHV	:
First and last name of the supervisor	:
Function of the supervisor	:
Contact of the supervisor	:

II. MANAGEMENT OF THE COMMUNITY SITE

#	Questions	Response Options
1	Site exists and is functional?	Yes/No
2	Case management of malaria for children under 5 years old?	Yes/No
3	CHV trained on the management of malaria in children under 5 years old in the last two years?	Yes/No
Availability of commodities		
1	Are cups available for the DOT (Directly Observed Therapy) strategy?	Yes/No
2	Soap or gel available?	Yes/No
3	Alcohol available?	Yes/No
4	Cotton available?	Yes/No
Availability of medicines		
1	ACT for children aged 2-11 months available?	Yes/No
2	ACT for children aged 1-5 years available?	Yes/No
3	mRDT available?	Yes/No
3	Paracetamol 500 mg Tablets available ?	Yes/No
4	Paracetamol 1000 mg Tablets available ?	Yes/No
5	Artesunate suppository available?	Yes/No

III. AVAILABILITY OF DOCUMENTS, DATA COLLECTION TOOLS, AND HEALTH PROMOTION TOOLS

#	Questions	Response Options
Availability of documents		

1	CHV guide	Yes/No
2	CHW manual	Yes/No
3	Children's health record cards	Yes/No
4	mRDT job aids	Yes/No
Availability of materials		
1	Timer	Yes/No
2	Mid-upper arm circumference (MUAC) tape	Yes/No
3	Scale	Yes/No
4	Calculator	Yes/No
5	Safety box	Yes/No
6	Garbage bin with lid	Yes/No
7	Garbage pit	Yes/No
Availability of data collection tools		
1	Children's consultation register	Yes/No
2	Health promotion register	Yes/No
3	Referral and counter-referral form	Yes/No
4	Stock and input register	Yes/No
5	Inventory form	Yes/No
6	Monthly report form	Yes/No
Availability of health promotion tools		
1	Awareness tools on malaria and integrated vector management	Yes/No
2	Megaphone	Yes/No

IV. COMPLETION OF DATA REPORTING TOOLS

#	Questions	Response Options	
Case management tools			
1	Intake form for children under 5 years old	Value (Total number of forms completed correctly)	Value (Total number of forms completed)
Reporting Tools			
1	Number of suspected malaria cases	Value reported in the register	
		Value reported in the monthly report of CHV activities	
2	Number of suspected malaria cases tested with mRDT	Value reported in the register	
		Value reported in the monthly report of CHV activities	
3	Number of uncomplicated malaria cases confirmed with mRDT	Value reported in the register	
		Value reported in the monthly report of CHV activities	
4	Number of uncomplicated malaria cases confirmed and treated with ACT	Value reported in the register	
		Value reported in the monthly report of CHV activities	

V. mRDT PRACTICE

#	Questions	Response Options
1	Did the CHV weigh the child?	Yes/No
2	Did the CHV perform the mRDT test to confirm malaria?	Yes/No
3	Did the CHV verify the expiration date of the mRDT?	Yes/No
4	Did the CHV was his/her hands with soap, dried them and put on	Yes/No

	gloves?	
5	Did the CHV verify the mRDT kits?	Yes/No
6	Did the CHV write the initials (name and date) of the child on the cassette?	Yes/No
7	Did the CHV clean the tip of the child's finger with alcohol swab and allowed it to dry?	Yes/No
8	Did the CHV press the tip of the child's finger to be punctured with his/her left hand and hold the lancet with his/her right hand?	Yes/No
9	Did the CHV puncture the finger without hesitation?	Yes/No
10	Did the CHV dispose of sharp objects in the security box?	Yes/No
11	Did the CHV wipe the first drop of blood with cotton?	Yes/No
12	Did the CHV collect an adequate amount of blood with the pipette?	Yes/No
13	Did the CHV ask the child's mother to apply pressure on the puncture site with dry cotton?	Yes/No
14	Did the CHV deposit blood in the correct well of the cassette?	Yes/No
15	Did the CHV dispose of the used pipette in the security box?	Yes/No
16	Did the CHV dispense the recommended number of drops of buffer in the designated area of the cassette?	Yes/No
17	Did the CHV remove and dispose of his gloves, transfer devices, and other blood-contaminated materials appropriately?	Yes/No
18	Did the CHV wait for the correct incubation time according to the manufacturer's instruction?	Yes/No

VI. MANAGEMENT OF ILLNESSES IN CHILDREN UNDER FIVE YEARS OF AGE

#	Questions	Response Options
Diagnostic		
1	Child less than 2 months old?	Yes/No
2	Did the CHV ask if the child was sick for 14 days or more?	Yes/No
3	Did the CHV verify if the mid-upper arm circumference is less than 125 mm?	Yes/No
4	Did the CHV verify if the child could not drink water or breastfeed?	Yes/No
5	Did the CHV verify if the child throw up everything he/she eats or drinks?	Yes/No
6	Did the CHV verify if the child has had a seizure?	Yes/No
7	Did the CHV verify the paleness of palms of the hands?	Yes/No
8	Is the child currently having seizures?	Yes/No
9	Did the CHV verify if the child is in a state of coma?	Yes/No
10	Did the CHV verify if the child presents with edema of the lower limbs (feet)?	Yes/No
11	Did the CHV verify if the child has an elevated temperature?	Yes/No
12	Given the child's positive mRDT results, did the CHV confirm uncomplicated malaria?	Yes/No
13	Given the child's positive mRDT results, did the CHV conclude the presence of a suspected severe malaria case?	Yes/No
14	Given the child's positive mRDT results, did the CHV check for signs of other diseases?	Yes/No

15	Given the child's negative test result, did the CHV conclude on the absence of malaria.	Yes/No
Treatment		
1	Did the CHV fill out line 7 of the intake form?	Yes/No
2	Did the CHV fill out the referral or counter-referral forms?	Yes/No
3	Did the CHV administer the first dose of treatment on site?	Yes/No
4	Did the CHV note the treatment and advice on line 8 (intake form)	Yes/No
5	Did the CHV use the job aid to explain the treatment to the child's mother?	Yes/No
6	Did the CHV correctly classify and count the medicine for the child based on his/her age group?	Yes/No
7	Did the CHV explain the dosage of the medicine to the child's mother?	Yes/No
8	Did the CHV verify that the child's mother understands the explanations given?	Yes/No
9	Did the CHV counsel the child's mother on nutrition?	Yes/No
10	Did the CHV counsel the child's mother on malaria (cause, risks, and prevention)?	Yes/No
11	Did the CHV invite the child's mother to return at any signs of complication?	Yes/No
12	Did the CHV refer the child with uncomplicated malaria confirmed with a positive mRDT to the attached health center?	Yes/No
13	Did the CHV refer the child with severe symptoms to the attached health center?	Yes/No

VII. SENSITIZATION ON INTEGRATED VECTOR MANAGEMENT

#	Questions	Response Options
1	Has the CHVs carried out awareness-raising and advocacy activities at the level of local authorities?	Yes/No
2	Has the CHVs carried out sensitization activities at the village level?	Yes/No
3	Report on sensitization available?	Yes/No
4	Quarterly sanitation report available?	Yes/No
Activities on integrated vector management (IVM)		
1	Sensitization	Yes/No
2	General sanitation	Yes/No
3	Backfilling standing water	Yes/No
4	Planting mosquito repellent trees	Yes/No
5	Stratification map of larval sites at village level available?	Yes/No
6	Are there preventive measures for people who travel frequently at the village level?	Yes/No

FINAL EVALUATION SCORES :

Level A: Score greater or equal to 80%

Level B: Score between 60% and 79%

Level C: Score less than 60 %

S5: Malawi CHW Supervision Checklist.

Excerpt from the Malawi CHW (known locally as HSAs) supervision checklist. Source: Malawi Ministry of Health.

**COMMUNITY CASE MANAGEMENT H.S.A SUPERVISION CHECKLIST**					
**Supervisor Name:**		**Date:**			
**HSA name:**		**District:**			
**Name of village clinic:**		**Health facility:**			
**Place where the clinic is operated:**		**Number of under five children:……….**			
**Distance from reporting H/F:**					
#	**Item**	**Yes**	**No**	**NA**	**Comment**
**A. CASE MANAGEMENT** (Observe the HSA managing a sick child, or use a case scenario from your supervision materials. **TICK** if you observed a **sick child** __ or if you used a **case scenario** __.)					
1	Takes child's identification (name AND age AND sex)?				
2	Assesses for all danger signs **correctly**				
3	Identifies danger sign(s) **correctly**				
4	Counts respiratory rate correctly (+/- 2 breaths)				
5	Decides to treat or refer child's illness **correctly**				
6	Gives **correct** treatment				
7	Demonstrates how to administer treatment **correctly**				
8	Counsels (correct messages on feeding, increased fluids and when to return)				
9	Explains how to administer medicine correctly				
10	Asks caregiver to repeat back how to administer medicine				
11	Asks caregiver to return for follow-up visit				
12	Refers if child has danger sign or condition he/she cannot treat				
13	Facilitates referral (provides referral slip AND first dose)				
	**If fever performs mRDT correctly**				
14	Observes FEFO				
15	Procedure explained to the caregiver				
16	Site selected, cleaned with alcohol swab and allowed to dry				
17	Collects adequate volume of blood				
18	Avoids excessive squizing				
19	Waits for correct time according to manufacture's instructions				
20	Reads the results **correctly**				
21	Records the results				
22	Disposses off waste materials in sharps and non sharps containers				
	**OVERALL SUMMARY ("Yes" for 2, 5, 6 and 8, if fever then including 16, 17, 19, and 20)**				

**E. AVAILABILITY OF MEDICINE** (Check medicines and ask about availability.)					
36	Do you have a medicine requisition order receipt (records of medicine recently ordered / received)				
37	Do you receive the required amount of medicines ordered				
38	Are quantities of medicines received the same on requisition book & on Form 1A?				
39	Amoxicilin (approximately 60 tablets)				
40	Did you have Amoxicilin everyday last month? If no, for about how many days were you without Amoxicilin?.........				
41	Rectal Artesunate (At least 10 suppositories)				
42	Did you have Artesunate everyday last month? If no, for how many days were without Artesunate?.......				
43	LA 1X6 (At least 36 tablets = 6 blister packs)				
44	LA 2X6 (At least 48 tablets = 4 blister packs)				
45	Did you have LA everyday last month? If no, for about how many days were you without LA last month?.........				
46	mRDT (At least 10 tests)				
47	Did you have mRDT everyday last month? If no, for about how many days were you without mRDT?.........				
48	ORS (At least 12 Sachets)				
49	Did you have ORS everyday last month? If no, for about how many days were you without ORS last month?............				
50	Zinc (Approximately 60 tablets)				
51	Paracetamol (Approximately 36 tablets)				
52	Eye ointment (At least 6 tubes)				
53	Did you have a continuous supply of LA, mRDT, RA, Amoxicilin, and ORS for the last 3 months without any stock-out of those products?				
54	Did you have a timer and a continuous supply of LA, mRDT, RA, Amoxicilin, ORS and zinc for the last 3 months without stock-out of any for 7 or more days?				

**F. AVAILIBILITY OF SUPPLIES** (Ask HSA to show you the following.)					
55	PPE for COVID-19 IPC: Face Masks/Shield				
	– Hand sanitizer				
	- Soap				
56	Disposable gloves				
57	Disposable Aprons				
58	Appropriate timer (measures seconds) available and functioning				
59	Blank Monthly Report forms (at least 2)				
60	Village Clinic Register with blank pages (for at least 10 cases)				
61	Laminated Sick Child Recording Form in color				
62	Blank referral slips (at least 3)				
63	Supervision Log Book				
64	MUAC tape				
65	Plastic pail				
66	Basin				
67	Spoons (at least 2)				
68	Cups (at least 2)				
69	Sharps containers				
70	Biohazard bags				
	**OVERALL SUMMARY ("Yes" for items 58, 59, 62 and 64)**				

S6: Madagascar CHV Supervision Checklist Data Visualization.

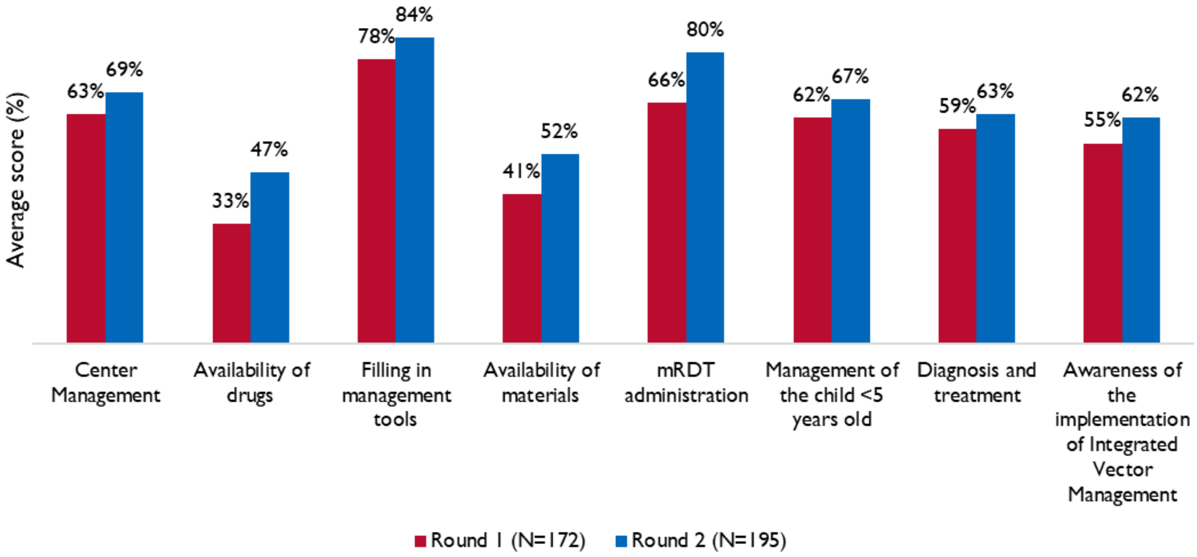

Example of how data collected through the Madagascar CHV supervision checklist is analyzed and visualized. Source: 2022 CHV supervision data, Faratsiho district, Vakinankaratra region, Madagascar.

S7: Mali CHW Supervision Checklist Data Visualization.

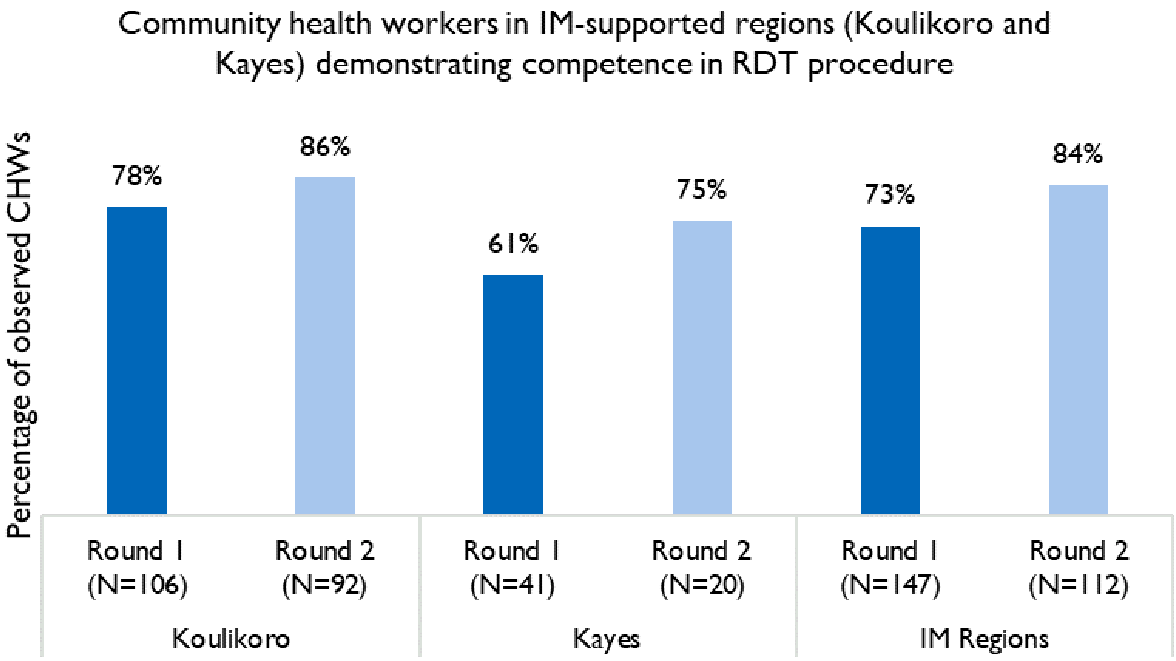

Figure 5. Example of how data collected through the Mali CHW supervision checklist is analyzed and visualized, Source: 2022 CHW supervision data, Koulikoro and Kayes regions, Mali.
